# New RAPMYCOI Sensititre^TM^ Antimicrobial Susceptibility Test for Atypical Rapidly Growing Mycobacteria (RGM)

**DOI:** 10.3390/diagnostics12081976

**Published:** 2022-08-15

**Authors:** Anna Borek, Anna Zabost, Agnieszka Głogowska, Dorota Filipczak, Ewa Augustynowicz-Kopeć

**Affiliations:** Department of Microbiology National Tuberculosis and Lung Diseases Research Institute, 01-138 Warsaw, Poland

**Keywords:** rapidly growing mycobacteria, antimicrobial resistance, broth microdilution, minimal inhibitory concentration (MIC)

## Abstract

Rapidly growing mycobacteria (RGM) cause an increasing international concern, mainly due to their natural resistance to many antibiotics. The aim of this study was to conduct species identification and determine the antimicrobial susceptibility profiles of RGM isolated in Poland. Antimicrobial susceptibility was tested using broth microdilution and the RAPMYCOI panel. A total of 60 strains were analysed, including the following species: *M. fortuitum* complex (30), *M. abscessus* subsp. *abscessus* (16), *M. abscessus* subsp. *massiliense* (7), *M. chelonae* (5), and *M. mucogenicum* (2). For 12 *M. abscessus* subsp. *abscessus* strains, the presence of the erm 41T28 genotype associated with inducible macrolide resistance and a functional *erm* gene was confirmed. A MUT2 mutation in the *rrl* gene (constitutive resistance) was identified for two strains from the subtype *M. abscessus* subsp. *massiliense*. Among the 15 tested antibiotics, amikacin and linezolid had the strongest antimycobacterial activity. Most of the tested strains were resistant to doxycycline and trimethoprim/sulfamethoxazole. Tigecycline MICs were low for all tested strains. Findings from our study highlight the importance of correct identification of clinical isolates and antimicrobial susceptibility testing.

## 1. Introduction

Nontuberculous mycobacteria (NTM), also known as mycobacteria other than tuberculosis (MOTT), are ubiquitous environmental microorganisms [1]. Currently, more than 150 species are known worldwide and many of them are increasingly recognized as important human pathogens [2,3]. Based on their growth rate, NTM species are divided into slowly growing mycobacteria (SGM) and rapidly growing mycobacteria (RGM) [4]. To date, more than 75 RGM species have been identified, which represents approximately 50% of all known mycobacterial species [5]. Due to advances in molecular research, the number of newly discovered species continues to increase.

RGM species are classified into six main taxonomic groups, distinguished based on genetic relatedness and the presence of pigment. These are:

(1) *M. fortuitum* (*M. fortuitum, M. peregrinum, M. sengalense, M. porcinum, M. neworleansense, M. boenickei, M. houstonense, M. brisbanense, M. septicum*, and *M. setense*),
(2) *M. chelonae/M. abscessus* complex (*M. chelonae, M. immunogenum, M. franklinii, M. salmoniphilum, M. abscessus* subsp. *abscessus, M. abscessus* subsp. *Massiliense,* and *M. abscessus* subsp. *bolletii*),
(3) *M. smegmatis* (*M. smegmatis* and *M. goodii*),
(4) *M. mucogenicum* (*M. mucogenicum*, *M. phocaicum*, and *M. aubagnense*),
(5) *M. mageritense/M. wolinskyi*,
(6) pigmented RGM species (*M. neoaurum, M. canariasense, M. cosmeticum, M. monacense,* and *M. bacteremicum*) [6,7,8,9,10,11,12].

Tuberculosis caused by *Mycobacterium tuberculosis* complex remains a serious global health problem in developing countries [13]. However, in recent decades, the number of reported cases of mycobacteriosis, a disease caused by atypical mycobacteria, has increased significantly [14]. Factors contributing to the increased incidence of NTM infections include: demographic changes, ageing of the population, increased incidence of comorbidities, and immunosuppression [15]. However, the epidemiology of NTM infections remains unknown as the reporting of mycobacterial cases to public health authorities is not mandatory in most countries [16]. Undoubtedly, the diagnosis of the disease is facilitated by recently improved testing methods [17].

Epidemiological statistics indicate that people living in Asia are particularly susceptible to NTM infections. In 2014, the incidence of NTM in the Japanese population was estimated at 14.7/100,000 [18,19,20]. In Great Britain, the incidence of NTM infection increased from 0.9 to 2.9/100,000 between 1995 and 2006 [21]. Studies from North America and Australia revealed that the annual incidence of NTM in these regions in 1997–2010 was 3.2–9.8/100,000 [14]. In Denmark, the incidence of NTM increased between 2003 and 2008 from 0.6 to 1.5/100,000 [22]. In Poland, statistics published by the National Institute of Public Health (PZH) and the Chief Sanitary Inspectorate show that the incidence rate of mycobacteriosis was 0.69 in 2017, 0.63 in 2018, and 0.61/100,000 in 2019 [23].

Atypical mycobacteria are opportunistic pathogens, ubiquitous in the environment, and are found in fresh and marine water, soil, and on biofilms [24]. Infections mainly concern the population of high-risk patients, which includes patients with cystic fibrosis (CF), bronchiectasis, emphysema, chronic obstructive pulmonary disease (COPD), and immunoincompetence (human immunodeficiency virus (HIV) infection, organ transplant, diabetes mellitus, renal failure). Among rapidly growing mycobacteria, the highly pathogenic non-pigmented species include *M. fortuitum, M. abscessus,* and *M. chelonae*, which are responsible for more than 80% of all clinical cases [25].

The clinical manifestations of RGM infections are very diverse. They most often concern the respiratory tract, skin, soft tissues, bones and joints, lymphadenitis, or disseminated infections [26]. Chronic lung infections are usually caused by *M. abscessus* subsp. abscessus and *M. abscessus* subsp. *massiliense* [25,27]. In patients with cystic fibrosis, these pulmonary infections are associated with a very high mortality. *M. fortuitum* complex is most frequently isolated from infected skin after accidental injuries, cosmetic procedures, and laser surgery. Reportedly, *M. abscessus* is responsible for 90% of respiratory diseases caused by RGM, and *M. fortuitum* is responsible for 60–80% of postsurgical and catheter-related infections [26]. The most common symptoms of infection caused by *M. chelonae* are diseases of the skin, bones, and soft tissues as well as ophthalmic infections, including keratitis. Rapidly growing mycobacteria are also isolated from patients with catheter-related bloodstream infection. In this case, the causative pathogens are *M. mucogenicum* and *M. fortuitum*, but also *M. neoaurum* and *M. bacteremicum* [28,29,30,31].

To determine the etiological factors of mycobacteriosis, it is necessary to correctly identify atypical mycobacteria to the species level. This is due to the different antimicrobial susceptibilities of mycobacteria. The management of a wide spectrum of NTM infections is a serious challenge worldwide. The selection of the appropriate antibiotic therapy for the patient should be based on the results of in vitro antimicrobial susceptibility testing.

However, the suitability of antimicrobial susceptibility testing in the treatment of patients with mycobacteriosis remains controversial due to the discrepancy between test results and clinical response [27,32]. Good correlations demonstrated in the studies carried out to date have been shown for two groups of antibiotics: macrolides and aminoglycosides.

Macrolides (clarithromycin and azithromycin) are among the basic antibiotics used in the treatment of mycobacteriosis. All macrolides bind to the V domain in 23S rRNA on the 50S ribosome subunit [33]. Two mechanisms of resistance to this class of drugs have been identified so far among atypical mycobacteria. The first mechanism is the constitutive resistance associated with a point mutation at either the A2058 or A2059 position of the 23S rRNA (*rrl* gene). The second mechanism, defined as inducible macrolide resistance, is associated with functional *erm* genes encoding ribosomal methyltransferase. The *erm* genes have been identified in the following species: *erm* (41) in *M. abscessus* subsp. *abscessus* (serovars I, VI, VII (80% of isolates)) and *M. abscessus* subsp. *bolletii*; *erm* (39) in *M. fortuitum*, *M*. *houstonense*, *M*. *porcinum,* and *M*. *neworleansense*; *erm* (38) in *M. smegmatis* and *M*. *goodie*; *erm* (40) in *M. mageritense* and *M. wolinskyi*. Clarithromycin-sensitive strains lack or have damaged *erm* genes. This group includes the following species: *M. abscessus* subsp. *abscessus* serovar II (Mab30), *M. abscessus* subsp. *massiliense*, *M. chelonae*, *M. immunogenum*, *M. mucogenicum group*, *M. peregrinum*, *and M. senegalense* [13,34,35].

The aminoglycosides (amikacin and tobramycin) act by binding stably to the 30S ribosomal subunit in bacterial cells, leading to misreading of the genetic code and inhibition of protein synthesis and consequently to cell death. Resistance to aminoglycosides in atypical mycobacteria is associated with single-point mutations in the 16S rRNA (*rrs* gene) [36].

According to the Clinical and Laboratory Standards Institute (CLSI), the broth microdilution method is considered the gold standard for testing the drug sensitivity of atypical RGM strains. Antimicrobial susceptibility testing should include the following antibiotics: clarithromycin, amikacin, moxifloxacin, linezolid, imipenem, cefoxitin, ciprofloxacin, doxycycline, trimethoprim/sulfamethoxazole, and tobramycin (only for *M. chelonae*). It is also recommended to determine the minimal inhibitory concentration (MIC) value for tigecycline, but to date there are no consensus breakpoints or guidelines for the interpretation of results [34,37].

There is a commercially available RAPMYCOI test for RGM from Thermo Fisher Scientific (Waltham, MA, USA) that includes all the antibiotics recommended for the treatment of RGM infections. *M. fortuitum* complex, *M. abscessus* subsp. *abscessus*, *M. abscessus* subsp. *massiliense* and *M. chelonae* are the most common rapidly growing mycobacteria (RGM) isolated in Poland.

In the presented study, the susceptibility of 60 RGM strains to 15 antibiotics was determined using the RAPMYCOI panels. The obtained results were compared with data published worldwide, which made it possible to obtain a complete picture of the drug resistance in this group of mycobacteria.

## 2. Materials and Methods

### 2.1. Bacterial Strains and Growth Conditions

The study was conducted on 60 strains of atypical mycobacteria (RGM) originally isolated from respiratory specimens (sputum, bronchial washings), in the period from 2019 to 2020 in mycobacterial laboratories in Poland.

The respiratory specimens were decontaminated with the sodium hydroxide and N-acetyl-L-cysteine (NaOH/NALC) (Chempur, Poland) method. The strains were cultured on solid media: egg-based Lowenstein-Jensen medium, Stonebrink medium, and in automated system MGIT (Becton Dickinson, Franklin Lakes, NJ, USA).

### 2.2. Strain Identification

For DNA extraction, the GenoLyse (Hain Lifescience, Nehren, Germany) kit was used according to protocol.

The strains were identified using the GenoType Mycobacterium CM assay ver. 2.0 (Hain Lifescience, Nehren, Germany) in accordance with the manufacturer’s instructions.

*Mycobacteria* from the *Mycobacterium abscessus* complex (MABC) were identified using the GenoType NTM-DR assay (Hain Lifescience, Nehren, Germany). *M. mucogenicum* was identified using the GenoType Mycobacterium AS assay (Hain Lifescience, Nehren, Germany).

The collection of RGM strains from patients with suspected tuberculosis included the following species: *M. abscessus* subsp. *abscessus* (16), *M. abscessus* subsp. *massiliense* (7), *M. fortuitum* complex (30), *M. chelonae* (5), and *M. mucogenicum* (2).

### 2.3. Molecular Determination of Antimicrobial Susceptibility to Macrolides and Aminoglycosides

GenoType NTM-DR assay enabled the detection of resistance to macrolides (*erm* (41) and *rrl* genes) and aminoglycosides (*rrs* genes).

*Erm* (41) gene was only detected in members of the *M. abscessus* complex.

The above test detected mutations at position 28 of the *erm* (41) gene:

- If the strain had a genotype in which C was at position 28 it meant that the tested strain was sensitive to macrolides.
- If the strain had a genotype in which T was at position 28 it meant that the tested strain was resistant to macrolides.

In the Table 1 and Table 2 below, the mutations detected by the applied test was shown.

### 2.4. Phenotypic Determination of Antimicrobial Susceptibility Profile

Antimicrobial susceptibility was tested using broth microdilution. For this purpose, 96-well RAPMYCOI Sensititre ™ titration plates (Thermo Fisher Scientific, Waltham, MA, USA) were used, which allow for the simultaneous determination of susceptibility to 15 antibiotics.

**Figure 1:**
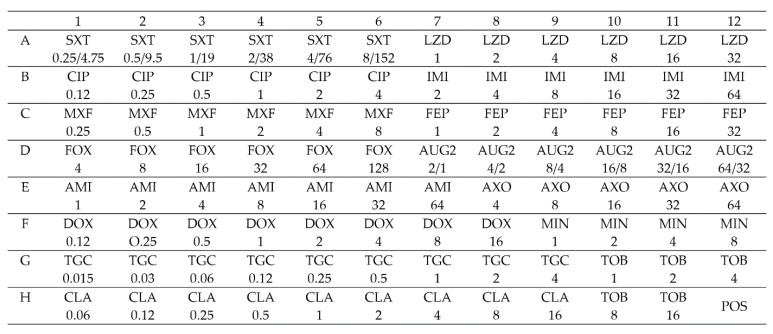
RAPMYCOI plate design: positive control (POS), amikacin (AMI), amoxicillin/clavulanic acid (AUG2), cefepime (FEP), cefoxitin (FOX), ceftriaxone (AXO), ciprofloxacin (CIP), clarithromycin (CLA), doxycycline (DOX), imipenem (IMI), linezolid (LZD), minocycline (MIN), moxifloxacin (MXF), trimethoprim/sulfamethoxazole (SXT), tigecycline (TGC), and tobramycin (TOB). The number under the antibiotic abbreviation shows its concentration in μg/mL.

RAPMYCOI plates contain freeze-dried antibiotics in a range of concentrations (μg/mL). The plate design and the tested antibiotic concentrations are presented in Figure 1.

At the first stage of the test, an inoculum of a mycobacterial suspension at the optical density of 0.5 McFarland scale was prepared. A total of 50 µL of inoculum was transferred to 10 mL of CAMHB medium (cation-supplemented Mueller-Hinton broth and TES buffer) (Thermo Fisher Scientific, Waltham, MA, USA). The 100 μL suspension prepared according to this protocol was pipetted onto a 96-well titration plate and incubated at 30 °C ± 2 °C. Plates with RGM were incubated for 3 to 5 days. Only for clarithromycin, the incubation period was prolonged to 14 days in order to detect inducible resistance associated with the presence of the *erm* genes. If microbial growth in the positive control sample was sufficient, MICs were measured. In cases of difficulties with visual reading, 10 µL of Alamar Blue (BIO-RAD, Hercules, CA, USA) reagent and 25 µL of 5% Tween 80 (Fisher Scientific, Hampton, NH, USA) were added. A colour change from blue to pink indicated the growth of a strain. Measured MICs were interpreted and each strain was classified into one of three groups (sensitive (S), intermediate (I), and resistant (R)) in accordance with the CLSI guidelines (document M62, 1st edition) (37) (Table 3).

## 3. Results

Table 4 below presents the percentage of strains that are sensitive, intermediate, and resistant to particular antibiotics. The classification was made on the basis of the obtained MIC values.

The data obtained in the performed antimycobacterial susceptibility test showed that amikacin and linezolid had the strongest antituberculotic activity against RGM. Most of the analysed strains were resistant to doxycycline and trimethoprim/sulfamethoxazole.

The following tables (Table 5, Table 6, Table 7, Table 8 and Table 9) present the obtained results separately for each RGM species.

### 3.1. Mycobacterium abscessus subsp. abscessus

All strains representing *Mycobacterium abscessus* subsp. *abscessus* were sensitive only to amikacin. Of the 16 strains, 12 (75%) were also sensitive to linezolid. However, they were all resistant to minocycline, trimethoprim/sulfamethoxazole, and doxycycline. Of the 16 strains, 13 (81%) were resistant to ciprofloxacin and moxifloxacin. Among the 16 strains from this subtype, 12 (75%) were clarithromycin-resistant (MIC > 16 µg/mL) (Table 5). The GenoType NTM-DR assay confirmed the presence of the functional *erm* (41) gene in these strains, associated with inducible macrolide resistance (erm41T28 genotype). Another four strains were sensitive to clarithromycin (erm41C28 genotype).

### 3.2. Mycobacterium abscessus subsp. massiliense

Strains representing *Mycobacterium abscessus* subsp. *massiliense* were sensitive to linezolid (100%) and amikacin (86%). They were all resistant to trimethoprim/sulfamethoxazole, doxycycline, and ciprofloxacin. Of the 7 strains, 2 (29%) representing the above subtype were resistant to clarithromycin (Table 6). The GenoType NTM-DR assay revealed the presence of the MUT2 mutation in the *rrl* gene (constitutive resistance) (Table 1). One strain representing *Mycobacterium abscessus* subsp. *massiliense* and sensitive to clarithromycin, with the MUT1 mutation in the *rrs* gene, was resistant to amikacin (MIC > 64 µg/mL) (Table 2).

### 3.3. Mycobacterium chelonae

All *Mycobacterium chelonae* strains (5) were sensitive to amikacin, clarithromycin, linezolid, and tobramycin, but resistant to trimethoprim/sulfamethoxazole, ciprofloxacin, and doxycycline (Table 7).

### 3.4. Mycobacterium mucogenicum

Two tested *Mycobacterium mucogenicum* strains (100%) were sensitive to clarithromycin, amikacin, cefoxitin, ciprofloxacin, moxifloxacin, and trimethoprim/sulfamethoxazole. One strain was resistant to doxycycline (MIC >16 µg/mL) (Table 8).

### 3.5. Mycobacterium fortuitum Complex

All strains representing *Mycobacterium fortuitum* complex (30) were sensitive to amikacin and moxifloxacin, 29 out of 30 strains were also sensitive to ciprofloxacin, 23 (77%) were resistant to clarithromycin, 17 (57%) were resistant to doxycycline, and 21 (70%) were resistant to trimethoprim/sulfamethoxazole (Table 9).

In the analysed collection of RGM, all 60 (100%) strains had low MIC values (from 0.06 to 1 µg/mL) for tigecycline, an antibiotic considered as a potential therapeutic agent and a drug of last resort in the treatment of severe cases of mycobacteriosis.

## 4. Discussion

With advances in molecular techniques and genetic tools, including whole genome sequencing (WGS), knowledge about the genetic diversity of NTM species and genes determining resistance to antibiotics continues to grow. Long treatment (18 to 24 months on average) and the need to use a combination of antibiotics with multiple side effects increase the importance of drug resistance testing, especially in RGM strains naturally resistant to first-line antituberculotic drugs.

Guidelines on antimicrobial susceptibility testing (AST) of atypical mycobacteria were developed by the CLSI and last updated in December 2018. Currently, CLSI M24 (3rd edition) provides recommendations on AST for slowly growing non-tuberculous mycobacteria, including *M. avium* complex (MAC), *M. kansasii*, and *M. marinum*, as well as rapidly growing mycobacteria (RGM) [34]. Since atypical mycobacteria may colonize the respiratory tract, their isolation from clinical specimens does not always correlate with the identification of an etiological factor responsible for the observed changes. This primarily refers to single sputum cultures. A negative sputum smear indicates a small number of microorganisms that are unlikely to be clinically significant, i.e., insufficient to establish a diagnosis of NTM. Therefore, detailed criteria for the diagnosis of mycobacteriosis have been developed for clinically significant isolates from the respiratory tract [27,38]. These criteria include the following:

- at least two NTM culture-positive sputa or one bronchial wash or lavage sample,
- a transbronchial or lung biopsy specimen with supporting mycobacterial histopathology and a positive NTM culture.

According to the current CLSI recommendations, AST includes antimicrobial agents for RGM such as amikacin, cefoxitin, ciprofloxacin, clarithromycin, doxycycline (or minocycline), imipenem, linezolid, moxifloxacin, trimethoprim-sulfamethoxazole, and tobramycin (for *M*. *chelonae* only) (Table 3). Worth noting is the fact that there are insufficient data to establish MIC breakpoints for tigecycline and clofazimine, and therefore for these agents a MIC without interpretation should be given [34].

The results of AST with selected drugs may concern specific species of atypical mycobacteria. For this reason, CLSI and most experts in RGM recommend identifying RGM strains at the species or even subspecies level (Table 10), especially for the *M*. *abscessus* complex, before performing a new AST RAPMYCOI and initiating treatment [5,34,39,40].

Because the incubation period for most RGM species ranges from 2 to 5 days, the final MIC reading in the RAPMYCOI test should be performed <5 days. This is mainly due to the instability of some drugs, including carbapenems and tetracyclines. There are only two exceptions where this incubation time should be extended when performing the RAPMYCOI test. The first case concerns strains representing *M. abscessus* complex isolated from patients who had a history of long-term treatment, including patients with cystic fibrosis. Mycobacterial strains isolated from this population of patients need a longer incubation period; therefore, in some cases it may be helpful to change the incubation temperature or to establish a culture in a shaking incubator. However, if the culture incubation period is longer than 5 days, results are only reliable for AST related to two drugs: clarithromycin and amikacin. The CLSI recommends a comment on the AST report such as: this NTM strain required extended incubation and results for only clarithromycin and amikacin are reliable after incubation for >5 days (Table 10) [34].

The second exception in the RAPMYCOI test to the incubation period longer than 5 days is clarithromycin. Phenotypical detection of inducible resistance to macrolides is achieved by extending the incubation of clarithromycin to 14 days unless the MIC is ≥16 μg/mL at an earlier time point. If the clarithromycin MIC is 4 or 8 μg/mL after 14 days of incubation, the test should be repeated. If the MIC is 4 or 8 μg/mL in the retest, sequencing of the *erm* gene for the given strain is recommended.

Worth noting is the fact that several RGM species have a non-functional or absent *erm* gene and are naturally sensitive to clarithromycin [41]. Therefore, sensitivity to clarithromycin can be reported at the initial MIC reading as no prolonged incubation is required for these specific species [42].

In the presented study, we identified 60 rapidly growing mycobacterial strains and determined their antimicrobial susceptibility in accordance with CLSI guidelines.

The most frequently isolated species was *Mycobacterium fortuitum* complex, which accounted for 50% (30/60) of all identified strains. The tests demonstrated that among all RGM species this group is characterized by high sensitivity to antibiotics. Our study confirmed this thesis and showed that 100% (30/30) of the strains from this group were sensitive to amikacin and moxifloxacin, 97% (29/30) were also sensitive to ciprofloxacin, and 93% (28/30) were sensitive to linezolid. In contrast, tests with clarithromycin showed a high level of resistance for 77% (23/30) of the strains. This resistance is higher compared to that reported by Sriram et al. (100% of sensitive strains among 30 tested) and Bhalla et al. (94.1% of sensitive strains among 17 tested) [43,44].

A low rate of drug resistance according to CLSI was also found for *Mycobacterium chelonae*. In our study, 100% of the strains (5/5) were sensitive to amikacin, clarithromycin, linezolid, and tobramycin. Our findings are consistent with those reported by Bhalla et al. In the cited study, no resistance to the four above-mentioned antibiotics was found for the three tested isolates [43].

*Mycobacterium chelonae* and *Mycobacterium mucogenicum* are classified into the group of species lacking functional *erm* genes. In our study, all strains of *M. chelonae* (5/5) and *M. mucogenicum* (2/2) were sensitive to clarithromycin and no *erm* genes were detected. However, Esteban et al. detected resistance to clarithromycin associated with the presence of *erm* genes in two strains of *M. chelonae* [45]. In a study by Davalos et al., 100% (2/2) of *M. chelonae* strains were sensitive to clarithromycin. However, one strain (25%) of *M. mucogenicum* resistant to this antibiotic was detected [46]. In our study, two strains representing *M. mucogenicum* were sensitive to most of the tested antibiotics. Only one strain was resistant to doxycycline and minocycline. A different antimicrobial susceptibility profile for this species was found by Faridah et al., who reported resistance to ciprofloxacin, doxycycline, clarithromycin, and tobramycin in a strain isolated from blood [47].

Isolates representing the *Mycobacterium abscessus* complex accounted for 38% (23/60) of all identified strains and it was the second largest group. Most strains (16) represented *M. abscessus* subsp. *abscessus* subtype, while *M. abscessus* subsp. *massiliense* subtype was less frequently identified (seven strains). We did not identify *M. abscessus* subsp. *bolletii*. In our study, most strains of *M. abscessus* subsp. *abscessus* (75%) were resistant to clarithromycin (MIC >16 µg/mL). This resistance was associated with the presence of a functional *erm* gene. The situation was different for *M. abscessus* subsp. *massiliense*. Only 28% of strains representing this subtype were clarithromycin-resistant, and the MUT2 mutation in the *rrl* gene was responsible for the resistance mechanism. Our findings confirm the worldwide reports on the more frequent resistance of *M. abscessus* subsp. *abscessus* to clarithromycin compared to *M. abscessus* subsp. *massiliense* [48,49,50]. Considering amikacin, the vast majority of strains were sensitive to this antibiotic. Only one strain (4%) was resistant to amikacin (MIC > 64 µg/mL) and had the MUT1 mutation in the *rrs* gene. Similar findings were reported by Bhalla et al., who found 92.3% of sensitive strains [43].

Among the three tested tetracyclines (doxycycline, minocycline, and tigecycline), the lowest MICs (from 0.06 to 1 µg/mL) were found for tigecycline. If we assume the criteria for interpretation proposed by Wallace et al. (resistant strain when MIC ≥ 8 µg/mL), all tested strains (60/60) were sensitive to tigecycline [51]. Similar relationships were observed by Pang et al.: sensitivity to tigecycline was found for 96% (53/55) of strains from the *M. abscessus* complex, 91% (10/11) of *M. fortuitum* strains, and 100% (3/3) of *M. chelonae* strains [52]. Similarly, in a study by Comba et al., the MIC value was <0.25 µg/mL for 45.7% of the strains (16/35), and from 0.25 µg/mL to 0.5 µg for 54.3% of the strains (19/35) [53]. According to worldwide reports, tigecycline is used in the treatment of the most severe infections with RGM mycobacteria, but to date there are no CLSI guidelines for the interpretation of MIC values in the AST.

## 5. Conclusions

The new RAPMYCOI test is a rapid tool for the determination of drug resistance profile in RGM. The obtained results are reliable and reproducible, and the test setup is not time-consuming. The broth microdilution method on which the test is based and the selection of antibiotics are consistent with the CLSI guidelines.

Taken together, the findings from the presented study highlight the importance of a correct identification of clinical isolates to the species and subtype level and the role of antimicrobial susceptibility testing, especially for highly resistant rapidly growing mycobacteria (RGM). The obtained results confirm previous assumptions published worldwide according to which there are predictable drug resistance profiles depending on the identified mycobacterial species. However, there are some exceptions to this rule, and therefore the drug resistance of individual strains should be tested as standard practice. The correlation between data obtained from AST with clinical findings proving the effectiveness of treatment will enable the development of new therapeutic regimens. As a result, effective drugs can be selected and the patient’s treatment optimized at an early stage.

## Figures and Tables

**Table 1:** Mutations determining resistance to macrolides detected using the GenoType NTM-DR assay within the *rrl* gene.

Absence of Wild-Type Band	Analysed Nucleic Acid Positions	Mutation Bands Present	Mutation	Phenotypic Resistance
*rrl* WT	2058–2059	*rrl* MUT1	A2058C	macrolides
		*rrl* MUT2	A2058G	
			A2058T	
		*rrl* MUT3	A2059C	
		*rrl* MUT4	A2059G	
			A2059T	

**Table 2:** Mutations determining resistance to aminoglycosides detected using the GenoType NTM-DR assay within the *rrs* gene.

Absence of Wild-Type Band	Analysed Nucleic Acid Positions	Mutation Bands Present	Mutation	Phenotypic Resistance
rrs WT	1406–1409	*rrs* MUT1	A1408G	aminoglycosides
			T1406A	
			C1409T	

**Table 3:** Antimicrobial agents and susceptibility breakpoints (MICs) for testing rapidly growing mycobacteria.

Antimicrobial Agent	MIC (μg/mL)			Comment
	S	I	R	
AMI	≤16	32	≥64	*M. abscessus* complex isolates with MIC of ≥64 μg/mL should be retested and/or the 16S rRNA gene sequenced to check for mutation
FOX	≤16	32–64	≥128	
CIP	≤1	2	≥4	Ciprofloxacin and levofloxacin are interchangeable, but both are less active than the newer B-methoxy-fluoroquinolones
CLA	≤2	4	≥8	See text for information on the *erm* gene; clarithromycin and azithromycin are interchangeable clinically
DOX	≤1	2–4	≥8	
MIN	≤1	2–4	≥8	
IMI	≤4	8–16	≥32	All isolates of *M. fortuitum*, *M. smegmatis*, and the *M. mucogenicum* group are presumed imipenem susceptible; imipenem MICs do not predict meropenem or ertapenem susceptibility
LZD	≤8	16	≥32	
MXF	≤1	2	≥4	
TMP-SMX	≤2/38		≥4/76	MIC is 80% inhibition
TOB	≤2	4	≥8	Predominantly for *M. chelonae*; if MIC >4 μg/mL, the test should be repeated and/or the identification confirmed by *rpo*β gene sequencing
TGC				Insufficient data to establish breakpoints; only MIC should be reported

**Table 4:** Classification of analysed RGM species into groups: (S)-sensitive, (I)-intermediate, and (R)-resistant, based on the measured MIC values.

	*M. abscessus* subsp. *abscessus* *n* = 16	*M. abscessus* subsp. *massiliense* *n* = 7	*M. chelonae* *n* = 5	*M. mucogenicum* *n* = 2	*M. fortuitum* complex *n* = 30
ANTIBIOTIC AGENT	values in (%)				
AMI	100 (S)	86 (S) 14 (R)	100 (S)	100 (S)	100 (S)
FOX	100 (I)	86 (I) 14 (S)	80 (S) 20 (R)	100 (S)	67 (I) 33 (S)
CIP	81 (R) 19 (I)	100 (R)	100 (R)	100 (S)	97 (S) 3 (I)
CLA	75 (R) 25 (S)	71 (S) 29 (R)	100 (S)	100 (S)	77 (R) 23 (S)
IMI	100 (I)	100 (I)	80 (R) 20 (I)	50 (S) 50 (I)	63 (I) 27 (S) 10 (R)
LZD	75 (S) 25 (I)	100 (S)	100 (S)	100 (S)	93 (S) 7 (I)
DOX	100 (R)	100 (R)	100 (R)	50 (S) 50 (R)	56,6 (R) 43,3 (S)
MIN	100 (R)	57 (S) 43 (R)	100 (R)	50 (S) 50 (I)	56,6 (R) 43,3 (S)
MXF	81 (R) 19 (I)	86 (R) 14 (I)	80 (R) 20 (S)	100 (S)	100 (S)
SXT	100 (R)	100 (R)	100 (R)	100 (S)	70 (R) 30 (S)
TOB			100 (S)		

**Table 5:** Results of in vitro susceptibility testing for *M. abscessus* subsp. *abscessus* strains.

	*M. abscessus* subsp. *abscessus* (*n* = 16)										
	AMI	FOX	CIP	CLA	DOX	IMI	LZD	MIN	MXF	TGC	SXT
1	4 (S)	32 (I)	2 (I)	0.5 (S)	>16 (R)	16 (I)	8 (S)	>8 (R)	4 (R)	0.5	>8/152 (R)
2	8 (S)	32 (I)	4 (R)	>16 (R)	16 (R)	16 (I)	≤1 (S)	>8 (R)	2 (I)	0.5	8/152 (R)
3	4 (S)	32 (I)	4 (R)	>16 (R)	>16 (R)	16 (I)	16 (I)	>8 (R)	4 (R)	0.5	>8/152 (R)
4	2 (S)	32 (I)	4 (R)	>16 (R)	>16 (R)	16 (I)	8 (S)	>8 (R)	4 (R)	0.5	>8/152 (R)
5	4 (S)	32 (I)	4 (R)	>16 (R)	>16 (R)	16 (I)	8 (S)	>8 (R)	4 (R)	0.12	>8/152 (R)
6	2 (S)	32 (I)	>4 (R)	>16 (R)	>16 (R)	8 (I)	8 (S)	>8 (R)	8(R)	0.5	>8/152 (R)
7	4 (S)	32 (I)	4 (R)	>16 (R)	>16 (R)	16 (I)	2 (S)	>8 (R)	4 (R)	0.06	8/152 (R)
8	4 (S)	32 (I)	2 (I)	2 (S)	>16 (R)	8 (I)	8 (S)	>8 (R)	4 (R)	1	8/152 (R)
9	8 (S)	32 (I)	4 (R)	1 (S)	>16 (R)	8 (I)	4 (S)	>8 (R)	4 (R)	0.25	>8/152 (R)
10	4 (S)	32 (I)	4 (R)	>16 (R)	>16 (R)	8 (I)	8 (S)	>8 (R)	4 (R)	0.25	>8/152 (R)
11	4 (S)	32 (I)	>4 (R)	>16 (R)	>16 (R)	16 (I)	16 (I)	>8 (R)	>8(R)	1	>8/152 (R)
12	8 (S)	32 (I)	>4 (R)	>16 (R)	>16 (R)	16 (I)	16 (I)	>8 (R)	>8 (R)	1	>8/152 (R)
13	4 (S)	32 (I)	2 (I)	>16 (R)	>16 (R)	16 (I)	2 (S)	>8 (R)	2 (I)	0.25	4/76 (R)
14	4 (S)	32 (I)	4 (R)	>16 (R)	>16 (R)	16 (I)	4 (S)	>8 (R)	2 (I)	0.25	>8/152 (R)
15	4 (S)	64 (I)	4 (R)	>16 (R)	>16 (R)	16 (I)	16 (I)	>8 (R)	8 (R)	0.5	>8/152 (R)
16	4 (S)	32 (I)	>4 (R)	0.12 (S)	>16 (R)	16 (I)	4 (S)	>8 (R)	4 (R)	0.25	>8/152 (R)

**Table 6:** Results of in vitro susceptibility testing for *M. abscessus* subsp. *massiliense* strains.

	*M. abscessus* subsp. *massiliense* (*n* = 7)										
	AMI	FOX	CIP	CLA	DOX	IMI	LZD	MIN	MXF	TGC	SXT
1	4 (S)	32 (I)	4 (R)	0.25 (S)	>16 (R)	16 (I)	8 (S)	>8 (R)	8 (R)	1	>8/152 (R)
2	>64 (R)	16 (S)	4 (R)	≤0.06 (S)	>16 (R)	16 (I)	2 (S)	>8 (R)	2 (I)	0.12	8/152 (R)
3	4 (S)	32 (I)	4(R)	0.12 (S)	8 (R)	16 (I)	8 (S)	2 (S)	4 (R)	0.25	>8/152 (R)
4	8 (S)	32 (I)	>4 (R)	0.25 (S)	>16 (R)	16 (I)	8 (S)	>8 (R)	>8 (R)	0.5	>8/152 (R)
5	4 (S)	32 (I)	4 (R)	>16 (R)	16 (R)	16 (I)	8 (S)	2 (S)	8 (R)	0.5	8/152 (R)
6	4 (S)	32 (I)	4 (R)	>16 (R)	16 (R)	16 (I)	8 (S)	2 (S)	8 (R)	0.5	8/152 (R)
7	8 (S)	32 (I)	>4 (R)	0.25 (S)	>16 (R)	16 (I)	8 (S)	2 (S)	>8 (R)	0.5	>8/152 (R)

**Table 7:** Results of in vitro susceptibility testing for *M. chelonae* strains.

	*M. chelone (n* = 5)											
	AMI	FOX	CIP	CLA	DOX	IMI	LZD	MIN	MXF	TGC	TOB	SXT
1	16 (S)	>128 (R)	4(R)	0.5 (S)	>16 (R)	16 (I)	4 (S)	>8 (R)	1 (S)	0.5	2 (S)	8/152 (R)
2	8 (S)	64 (I)	4(R)	≤0.06 (S)	>16 (R)	32 (R)	4 (S)	>8 (R)	4(R)	0.25	≤1 (S)	8/152 (R)
3	4 (S)	64 (I)	4(R)	0.25 (S)	>16 (R)	32 (R)	4 (S)	>8 (R)	4(R)	0.5	≤1 (S)	>8/152 (R)
4	4 (S)	64 (I)	4(R)	0.25 (S)	>16 (R)	32 (R)	4 (S)	>8 (R)	4(R)	0.5	≤1 (S)	8/152 (R)
5	8 (S)	64 (I)	4(R)	0.25 (S)	>16 (R)	64(R)	4 (S)	>8 (R)	4(R)	0.25	≤1 (S)	>8/152 (R)

**Table 8:** Results of in vitro susceptibility testing for *M. mucogenicum* strains.

	*M. mucogenicum* (*n* = 2)										
	AMI	FOX	CIP	CLA	DOX	IMI	LZD	MIN	MXF	TGC	SXT
1	2 (S)	16 (S)	0.25 (S)	0.25 (S)	>16 (R)	8 (I)	2 (S)	>8 (R)	≤0.25 (S)	0.25	1/19 (S)
2	≤1 (S)	8 (S)	0.5 (S)	0.12 (S)	≤0.12 (S)	4 (S)	2 (S)	≤1 (S)	0.5 (S)	0.12	0.5/9.5 (S)

**Table 9:** Results of in vitro susceptibility testing for *M. fortuitum* complex strains.

	*M. fortuitum* Complex (*n* = 30)										
	AMI	FOX	CIP	CLA	DOX	IMI	LZD	MIN	MXF	TGC	SXT
1	≤1 (S)	16 (S)	0.5 (S)	0.5 (S)	0.5 (S)	≤2 (S)	4 (S)	≤1 (S)	≤0.25 (S)	0.12	2/38 (S)
2	≤1 (S)	16 (S)	≤0.12 (S)	0.12 (S)	>16 (R)	≤2 (S)	2 (S)	>8 (R)	≤0.25 (S)	0.5	4/76 (R)
3	≤1 (S)	32 (I)	≤0.12 (S)	>16 (R)	>16 (R)	4 (S)	2 (S)	>8 (R)	≤0.25 (S)	0.25	0.5/9.5 (S)
4	≤1 (S)	16 (S)	≤0.12 (S)	0.12 (S)	>16(R)	4 (S)	≤1 (S)	>8 (R)	≤0.25 (S)	0.25	1/19 (S)
5	≤1 (S)	16 (S)	≤0.12 (S)	>16 (R)	≤0.12 (S)	8 (I)	≤1 (S)	≤1 (S)	≤0.25 (S)	0.25	2/38 (S)
6	≤1 (S)	32 (I)	≤0.12 (S)	>16 (R)	>16 (R)	8 (I)	16 (I)	>8 (R)	≤0.25 (S)	0.5	8/152 (R)
7	≤1 (S)	16 (S)	0.25 (S)	>16 (R)	0.5 (S)	4 (S)	≤1 (S)	≤1 (S)	≤0.25 (S)	0.25	1/19 (S)
8	≤1 (S)	16 (S)	≤0.12 (S)	0.25 (S)	>16(R)	4 (S)	≤1 (S)	>8 (R)	≤0.25 (S)	0.25	1/19 (S)
9	≤1 (S)	64 (I)	2(I)	>16 (R)	0.5 (S)	64(R)	8 (S)	>8 (R)	1 (S)	1	>8/152 (R)
10	≤1 (S)	64 (I)	0.25 (S)	>16 (R)	>16 (R)	16 (I)	8 (S)	>8 (R)	≤0.25 (S)	0.25	>8/152 (R)
11	2 (S)	64 (I)	0.25 (S)	>16 (R)	>16 (R)	16 (I)	8 (S)	>8 (R)	0.5 (S)	0.25	4/76 (R)
12	4 (S)	64 (I)	0.25 (S)	>16 (R)	0.25 (S)	64 (R)	4 (S)	≤1 (S)	≤0.25 (S)	0.06	2/38 (S)
13	≤1 (S)	32 (I)	≤0.12 (S)	>16 (R)	≤0.12 (S)	4 (S)	4 (S)	≤1 (S)	≤0.25 (S)	0.03	0.5/9.5 (S)
14	≤1 (S)	32 (I)	0.25 (S)	>16 (R)	>16 (R)	8 (I)	8 (S)	>8 (R)	≤0.25 (S)	0.25	8/152 (R)
15	≤1 (S)	32 (I)	≤0.12 (S)	>16 (R)	>16 (R)	8 (I)	8 (S)	>8 (R)	≤0.25 (S)	0.03	2/38 (S)
16	≤1 (S)	8 (S)	≤0.12 (S)	>16 (R)	8 (R)	4 (S)	2 (S)	≤1 (S)	≤0.25 (S)	0.12	4/76 (R)
17	≤1 (S)	32 (I)	0.25 (S)	>16 (R)	>16 (R)	8 (I)	8 (S)	>8 (R)	≤0.25 (S)	0.25	>8/152(R)
18	≤1 (S)	32 (I)	0.25 (S)	16 (R)	>16 (R)	8 (I)	8 (S)	>8 (R)	≤0.25 (S)	0.03	>8/152(R)
19	≤1 (S)	32 (I)	0.25 (S)	16 (R)	>16 (R)	8 (I)	8 (S)	>8 (R)	≤0.25 (S)	0.06	>8/152(R)
20	≤1 (S)	32 (I)	≤0.12 (S)	>16 (R)	0.25 (S)	16 (I)	4 (S)	≤1 (S)	≤0.25 (S)	0.06	4/76 (R)
21	≤1 (S)	32 (I)	≤0.12 (S)	>16 (R)	>16(R)	8 (I)	2 (S)	>8(R)	≤0.25 (S)	0.12	4/76 (R)
22	≤1 (S)	32 (I)	0.25 (S)	>16 (R)	0.5 (S)	8 (I)	8 (S)	≤1 (S)	≤0.25 (S)	0.5	>8/152(R)
23	≤1 (S)	32 (I)	≤0.12 (S)	>16 (R)	0.12 (S)	8 (I)	4 (S)	≤1 (S)	≤0.25 (S)	0.25	4/76 (R)
24	≤1 (S)	32 (I)	≤0.12 (S)	>16 (R)	0.25 (S)	8 (I)	4 (S)	≤1 (S)	≤0.25 (S)	0.25	8/152 (R)
25	≤1 (S)	16 (S)	0.5 (S)	1 (S)	0.25 (S)	8 (I)	8 (S)	≤1 (S)	≤0.25 (S)	0.25	4/76 (R)
26	≤1 (S)	32 (I)	≤0.12 (S)	>16 (R)	>16 (R)	16 (I)	16 (I)	>8 (R)	≤0.25 (S)	0.25	4/76 (R)
27	≤1 (S)	32 (I)	0.5 (S)	0.25 (S)	0.25 (S)	32R	8 (S)	≤1 (S)	≤0.25 (S)	0.25	4/76 (R)
28	≤1 (S)	16 (S)	≤0.12 (S)	>16 (R)	0.25 (S)	16 (I)	4 (S)	≤1 (S)	≤0.25 (S)	0.25	8/152 (R)
29	≤1 (S)	16 (S)	≤0.12 (S)	0.12 (S)	16 (R)	8 (I)	8 (S)	8 (R)	≤0.25 (S)	0.12	4/76 (R)
30	≤1 (S)	32 (I)	≤0.12 (S)	>16(R)	>16(R)	16 (I)	8 (S)	>8(R)	≤0.25 (S)	0.06	>8/152 (R)

**Table 10:** Interpretation of AST results for *M. abscessus* complex and clarithromycin.

Sensitivity to Clarithromycin on Days 3–5 of Incubation	Sensitivity to Clarithromycin on Day 14 of Incubation	Genetic Mechanisms	Subspecies of *M. abscessus*	Phenotypic Sensitivity to Macrolides
sensitive	sensitive	non-functional *erm* gene (41)	*M. a. massiliense*	sensitive to macrolides
sensitive	resistant	functional *erm* gene (41)	*M. a. abscessus* *M. a. bolletii*	inducible resistance to macrolides
resistant	resistant	23S point mutation in rRNA	any of the above listed	high constitutive resistance to macrolides

## Data Availability

Data supporting reported results can be found in source data collected in National Tuberculosis and Lung Diseases Research Institute.
